# Long‐Term Epidemiological Insights Into Human Cystic Echinococcosis in Northeastern Iran: A 17‐Year Retrospective Analysis

**DOI:** 10.1155/japr/8605086

**Published:** 2025-12-26

**Authors:** Seyed-Sajjad Alavi-Kakhki, Mohammad Ghorbani, Seyed-Reza Mirbadie, Milad Badri, Mohammad-Reza Rezaiemanesh, Nooshin Hashemi, Zahra Jabalameli, Ali Gholizadeh, Mohammad-Ali Mohaghegh

**Affiliations:** ^1^ Student Research Committee, Torbat Heydariyeh University of Medical Sciences, Torbat Heydariyeh, Iran, thums.ac.ir; ^2^ Department of Epidemiology, School of Health, Shiraz University of Medical Sciences, Shiraz, Iran, sums.ac.ir; ^3^ Health Sciences Research Center, Torbat Heydariyeh University of Medical Sciences, Torbat Heydariyeh, Iran, thums.ac.ir; ^4^ School of Medicine, Shahroud University of Medical Sciences, Shahroud, Iran, shmu.ac.ir; ^5^ Medical Microbiology Research Center, Qazvin University of Medical Sciences, Qazvin, Iran, qums.ac.ir; ^6^ Department of Laboratory Sciences, School of Paramedical Sciences, Torbat Heydariyeh University of Medical Sciences, Torbat Heydariyeh, Iran, thums.ac.ir; ^7^ School of Medicine, Bam University of Medical Sciences, Bam, Iran, mubam.ac.ir; ^8^ Vice Chancellery of Education and Research, Torbat Heydariyeh University of Medical Sciences, Torbat Heydariyeh, Iran, thums.ac.ir; ^9^ Department of Medical Laboratory Sciences, Faculty of Allied Medicine, Gonabad University of Medical Sciences, Gonabad, Iran, gmu.ac.ir

**Keywords:** echinococcosis, epidemiology, Iran, surgery

## Abstract

**Background:**

Cystic echinococcosis (CE) remains an important public health challenge in endemic regions of Iran, especially in areas with intensive livestock farming and close human–animal contact. Long‐term epidemiological analyses are essential for identifying patterns, informing control strategies, and evaluating intervention outcomes.

**Methods:**

A 17‐year cross‐sectional study design based on retrospective data (2006–2022) was conducted on surgically confirmed human CE cases from two referral hospitals in Razavi Khorasan Province, northeastern Iran. Demographic, clinical, and spatial data from patients residing in Torbat‐e Heydariyeh, Zaveh, Mahvelat, and Roshtkhar were reviewed. Descriptive and inferential statistical analyses were performed using SPSS v.25, including chi‐square, Mann–Whitney *U*, and one‐way ANOVA tests (*α* = 0.05). Temporal trends were assessed using Poisson regression, and case distribution was visualized via GIS‐based heatmapping (ArcGIS Pro 3.2).

**Results:**

A total of 232 CE surgical cases were recorded during a 17‐year period, corresponding to an average annual incidence of 13.6 and a surgical incidence rate of 3/100,000 population. The liver was the most affected organ (81.5%), and abdominal pain was the most frequent presenting symptom (73.7%). Two age peaks were observed at 21–40 and ≥ 61 years. Females (57.8%) and urban residents (64.7%) comprised the majority of patients. Regression analysis indicated a nonsignificant declining trend over time (*β* = −0.95, 95% CI: −2.71–0.81, *p* = 0.26). A statistically significant association was found between residency and organ involvement in patients with CE (*χ* ^2^ = 5.78, df = 1, *p* = 0.016).

**Conclusion:**

This 17‐year analysis reveals persistent CE burden in northeastern Iran despite a modest decline in recent years. Sustained One Health surveillance, public education, and strengthened veterinary–human collaboration are required to mitigate disease transmission and improve control outcomes.

## 1. Introduction

Hydatid cyst, also known as cystic echinococcosis (CE), is a zoonotic parasitic disease caused by the larval stage of the tapeworm *Echinococcus granulosus*. This parasite primarily affects domestic animals such as sheep, cattle, goats, and pigs, with dogs serving as its definitive hosts [1, 2]. In humans, many CE infections remain asymptomatic or undetected for extended periods. However, CE can lead to the formation of slow‐growing, potentially dangerous cysts in critical organs, most notably the liver and lungs [3]. CE is transmitted to humans via ingestion of parasite eggs, which can be found on contaminated food, water, or vegetables [4]. Following ingestion, the larvae hatch in the intestines and can migrate to almost any organ, though they most frequently localize in the liver (50%–70%) and lungs [5, 6]. The symptoms and severity of liver hydatid cysts depend on the size and location of the cystic structures. Early‐stage cysts are often asymptomatic but can become symptomatic as they grow or become complicated. Symptoms in such cases may include upper right abdominal pain, jaundice, nausea, vomiting, and abnormal liver function tests [7, 8].

Human hydatidosis presents significant public health challenges, causing considerable morbidity, mortality, and economic burdens to affected communities [9]. CE can cause serious complications such as cyst rupture, anaphylaxis, secondary bacterial infection, and biliary obstruction [10]. Surgical intervention remains the mainstay of treatment in many endemic areas, yet recurrence and postoperative complications remain substantial clinical issues [11, 12]. These aspects underscore the need for a better understanding of CE′s clinical characteristics and epidemiological patterns in endemic regions [13].

CE has a global distribution, with an incidence rate of 1–200 per 100,000 worldwide [14]. A recent systematic review and meta‐analysis estimated the overall seroprevalence of CE in Iran at 4.2% among human hosts [15]. According to financial analyses, the annual economic burden of CE in Iran exceeds $230 million, whereas the yearly incidence of CE‐related surgeries is estimated at 0.8–1.73 per 100,000 individuals [6, 16].

Despite its importance, epidemiological data on CE in northeastern Iran, particularly the Torbat‐e Heydariyeh district remain scarce. This scarcity of long‐term data limits understanding of temporal trends, the effectiveness of control measures, and local disease dynamics, which are essential for informing both national and international CE control strategies. Torbat Heydariyeh University of Medical Sciences serves as a referral hub for the city and its surrounding rural areas, covering an estimated population of approximately 455,000 people in Razavi Khorasan Province. The two university‐affiliated hospitals in this district manage most surgical CE cases in the region, making them representatives of the local epidemiological and clinical burden.

Therefore, this 17‐year retrospective study is aimed at assessing the epidemiology and clinical characteristics of CE in northeastern Iran, focusing on the Torbat Heydariyeh district, to provide a comprehensive understanding of the regional burden of this neglected zoonotic disease and inform future control strategies. By examining this extended period in a high‐risk area, the study also sheds light on the persistence and transmission dynamics of the disease, generating knowledge relevant to both regional policy‐making and the global epidemiological context of CE.

## 2. Material and Methods

### 2.1. Study Area

This study was conducted in four districts of Khorasan Razavi Province, northeastern Iran—Torbat Heydariyeh (3751.39 km^2^), Zaveh (2465.6 km^2^), Mahvelat (3043.99 km^2^), and Roshtkhar (4315.11 km^2^)—covering approximately 13,576 km^2^. The region lies within a semiarid climatic zone characterized by hot summers and cold winters, with annual precipitation ranging from 200 to 300 mm. The local economy relies heavily on agriculture and livestock farming, which facilitates the transmission cycle of *E. granulosus* through close interaction between dogs and livestock. Torbat Heydariyeh, the largest district and a regional hub, hosts two tertiary referral hospitals—9‐Dey and Razi Hospitals—that provide specialized surgical care for CE patients referred from surrounding rural and urban areas. These facilities serve an estimated catchment population of 455,000 people, encompassing both pastoral and urban communities. The combination of extensive livestock raising, traditional slaughtering practices, and growing urbanization makes this area a relevant setting for studying CE epidemiology.

### 2.2. Study Design and Data Collection

A cross‐sectional study design based on retrospective data was applied to review all surgically confirmed human CE cases registered between January 2006 and December 2022 in the two referral hospitals. A confirmed case of CE was defined as a patient who underwent surgical intervention with intraoperative identification of hydatid cyst(s), supported by either histopathological confirmation or characteristic imaging findings consistent with CE. Only cases meeting these criteria were included in the analysis. This study was approved by the Ethics Committee of Torbat Heydariyeh University of Medical Sciences (Ethical Code: IR.THUMS.REC.1398.028). Informed consent was waived due to the retrospective design and use of anonymized patient data, in accordance with local regulations and ethical guidelines.

Case retrieval was performed through hospital archives using International Classification of Diseases (ICD‐10) codes B67.0–B67.9, corresponding to echinococcosis by organ site. For each confirmed surgical case of CE, the following data were recorded:

Demographic variables (age, gender, residency [urban/rural], marital status, and year diagnosis), as well as clinical and surgical variables (cyst location, presence of multiple‐organ involvement, clinical presentation, hospital stay duration, surgical method [open or laparoscopic], recurrence, and postoperative albendazole regimen including dose and duration), were collected. Two trained nurses independently extracted the patient data using a study‐specific standardized form, and any inconsistencies were reviewed and resolved by a senior infectious disease specialist. Incomplete or ambiguous records were excluded from analysis.

Population data for the study region were obtained from the Statistical Center of Iran (national census reports) (https://www.amar.org.ir). Because official population figures are available only for census years, the mean population of the study region during the 17‐year study period was used as the denominator for calculating the overall incidence rate. Annual incidence rates per 100,000 population were calculated by dividing the number of surgical CE cases in each year by the corresponding census or estimated population and multiplying by 100,000.

### 2.3. Statistical Analysis

Statistical analyses were performed using SPSS software (Version 25.0, Chicago, Illinois, United States). Continuous variables were summarized as mean ± standard error of the mean (SEM) or median (interquartile range), depending on the distribution tested by the Kolmogorov–Smirnov test. Categorical variables were presented as frequencies and percentages.

Comparisons between categorical variables (e.g., gender vs. organ involvement, residency vs. cyst site) were performed using the chi‐square test or Fisher′s exact test as appropriate. Continuous variables were compared using Student′s *t*‐test, one‐way ANOVA, or Mann–Whitney *U* test. Statistical significance was defined as *p* < 0.05.

To assess temporal changes in the annual number of CE cases, a Poisson regression model was applied, with year as the independent variable and annual case count as the dependent variable. The regression coefficient (*β*) and 95% confidence intervals were calculated to determine the direction and magnitude of trends.

A multivariate logistic regression model was also fitted to identify independent predictors of hepatic involvement, including gender, age, marital status, and residency as covariates. Results are presented as odds ratios (ORs) with corresponding 95% CIs.

### 2.4. Spatial Analysis

The spatial distribution and clustering of CE cases were examined using ArcGIS Pro 3.2. Case frequencies were geocoded by district of residence. A heatmap was generated to visualize concentration gradients of CE occurrence across the study area. This approach enabled the identification of potential geographic hotspots and supported spatial interpretation of disease dynamics.

## 3. Results

### 3.1. Geographical Distribution of Surgical CE Cases

A total of 232 surgical cases of CE were diagnosed and treated in the two main referral hospitals during the 17‐year period from 2006 to 2022. This corresponds to an average of 13.6 surgical cases per year, with an annual incidence rate of 3 per 100,000 population.

**Figure 1:**
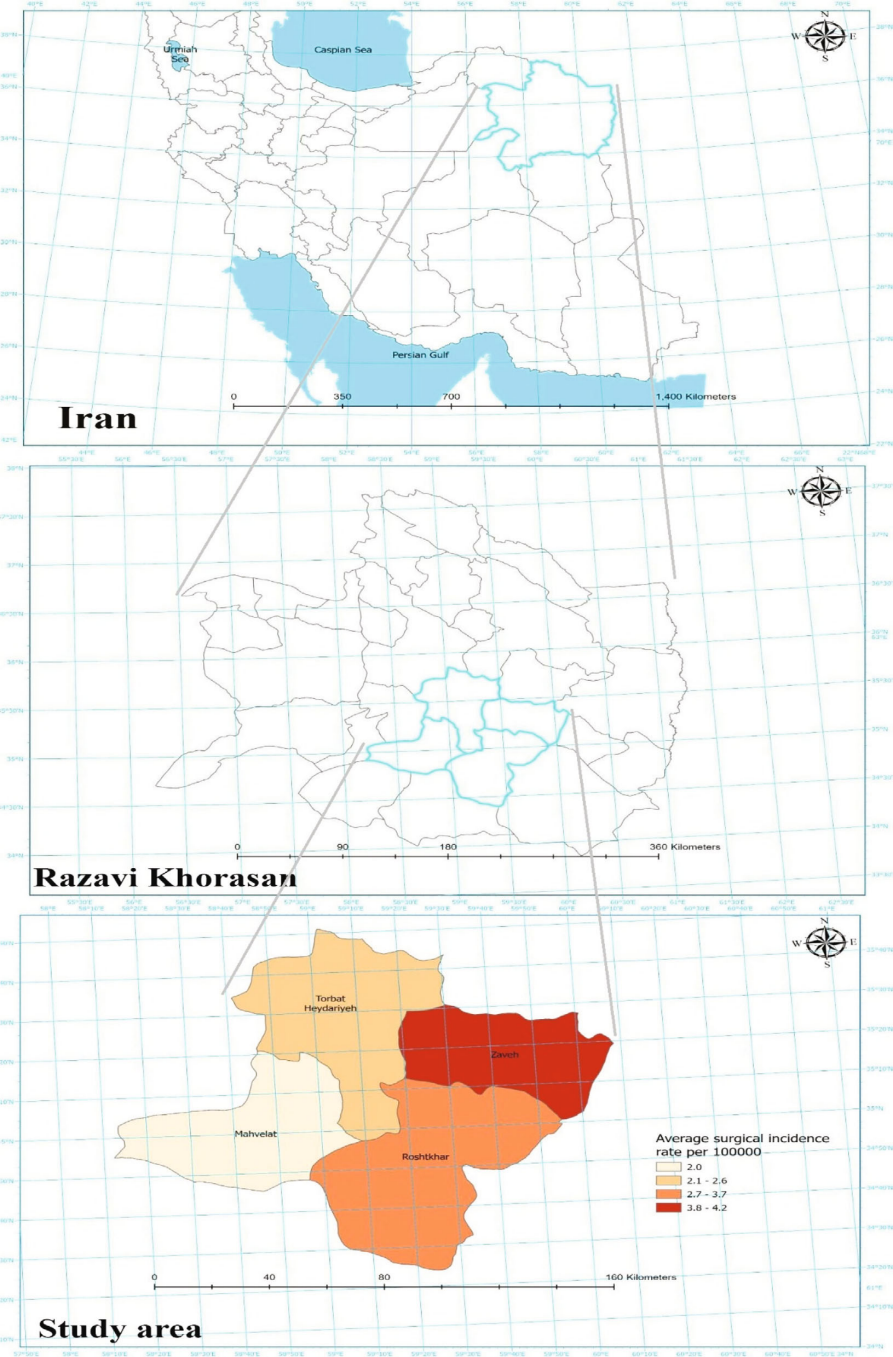
Geographic distribution and heatmap of surgical cystic echinococcosis incidence in counties of Razavi Khorasan Province, Iran.

The highest annual incidence was observed in Zaveh (4.2 per 100,000), followed by Roshtkhar (3.7 per 100,000), Torbat Heydariyeh (2.6 per 100,000), and Mahvelat (2.0 per 100,000) (Figure 1).

### 3.2. Temporal Trends

**Figure 2:**
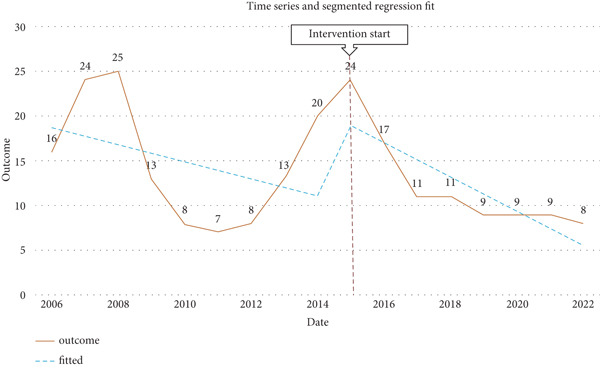
Time trend of surgically confirmed cystic echinococcosis cases (2006–2022) with segmented Poisson regression fit.

The annual number of surgical CE cases showed marked fluctuations during the 17‐year period (Figure 2). Peaks were observed in 2007 (*n* = 24), 2008 (*n* = 25), and 2015 (*n* = 24), followed by a gradual decline and stabilization after 2017. Regression analysis indicated a nonsignificant declining trend over time (*β* = −0.95, 95% CI: −2.71–0.81, *p* = 0.26). The mean annual number of cases in the post‐2017 period was approximately nine per year, compared with 17 per year during 2006–2016, representing a relative reduction of nearly 47%.

### 3.3. Demographic Characteristics

Of the 232 patients, 141 (60.8%) were female and 91 (39.2%) males, with a female‐to‐male ratio of 1.55:1. The mean age of patients was 46.27 ± 20.64 years, ranging from 3 to 93 years.

A bimodal age distribution was observed, peaking in the 21–40 years group (*n* = 84, 36.2%) and in those ≥ 61 years (*n* = 68, 29.3%).

A higher number of females was observed in both age groups, particularly among urban residents. Regarding residency, 150 patients (64.7%) were urban dwellers and 82 (35.3%) resided in rural areas (Table 1).

**Table 1:** Distribution of cystic echinococcosis cases by residency status, age group, and organ involvement in northeastern Iran (2006–2022).

**Residency**	**Age group**	**Liver,** **n** **(%)**	**Other organs,** **n** **(%)**	**Total,** **n** **(%)**
Urban	< 20	11 (100)	0 (0)	11 (100)
	20–40	45 (84.9)	8 (15.1)	53 (100)
	40–60	31 (88.6)	4 (11.4)	35 (100)
	> 60	42 (82.4)	9 (17.6)	51 (100)

Rural	< 20	4 (30.8)	9 (69.2)	13 (100)
	20–40	23 (74.2)	8 (25.8)	31 (100)
	40–60	19 (90.5)	2 (9.5)	21 (100)
	> 60	14 (82.4)	3 (17.6)	17 (100)

### 3.4. Surgical Management and Outcomes

**Figure 3:**
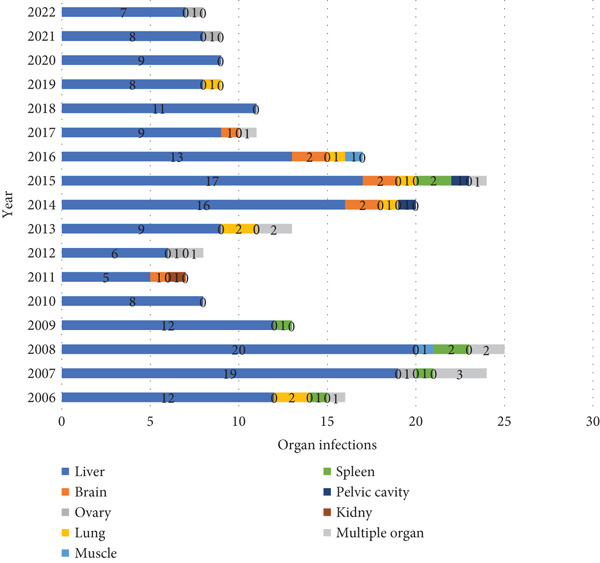
Distribution of cystic echinococcosis in different organs of surgical patients in northeastern Iran (2006–2022).

The liver was the most commonly affected organ (*n* = 189, 81.5%), followed by the lung (*n* = 8, 3.4%), brain (*n* = 8, 3.4%), and spleen (*n* = 7, 3.0%). Multiple‐organ involvement occurred in 11 patients (4.7%). Abdominal pain was the predominant presenting symptom (*n* = 182, 78.4%), followed by cough in pulmonary cases (*n* = 8, 3.4%) (Table 2). Although CE was not restricted to a single organ each year, in 2010, 2018, and 2020, all recorded cases were exclusively hepatic. In 2015, CE was reported in 24 patients, involving at least six different organ sites. Figure 3 illustrates the organ‐specific distribution of CE.

**Table 2:** Anatomical distribution of cystic echinococcosis and corresponding chief complaints in surgical patients in northeastern Iran (2006–2022).

**Location of cysts**	**No. (%)**	**Chief complains (no.)**
Liver	189 (81.5)	Abdominal pain (171)
		Abdominal pain and flank pain (10)
		Abdominal pain and jaundice (5)
		Abdominal pain, weakness, and itching (3)
Lung	8 (3.4)	Chest pain (2)
		Chest pain, cough, phlegm, and fever (6)
Brain	8 (3.4)	Headache and nausea (7)
		Seizure and fever (1)
Ovary	4 (1.72)	Abdominal pain (1)
		Abdominal pain and flank pain (3)
Muscle	2 (0.87)	Abdominal pain (1)
		Chest pain and weakness (1)
Spleen	7 (3)	Abdominal pain (4)
		Abdominal pain and flank pain (1)
		Abdominal pain, weakness, and itching (2)
Pelvic cavity	2 (0.87)	Abdominal pain (1)
		Pelvic pain (1)
Kidney	1 (0.43)	Dysuria and flank pain (1)
Liver and lung	5 (2.2)	Abdominal pain and flank pain (2)
		Chest pain, cough, phlegm, and fever (2)
		Abdominal pain, weakness, and itching (1)
Liver and spleen	1 (0.43)	Abdominal pain, weakness, and itching (1)
Liver and kidney	1 (0.43)	Abdominal pain and jaundice (1)
Liver and peritoneal cavity	1 (0.43)	Abdominal pain (1)
Liver and pelvic cavity	1 (0.43)	Abdominal pain (1)
Liver and sub diaphragm	1 (0.43)	Abdominal pain (1)
Spleen and peritoneal cavity	1 (0.43)	Jaundice, nausea, and anorexia (1)

The mean hospitalization duration was 8.4 ± 2.6 days, and postoperative albendazole therapy (10–15 mg/kg/day for 3 months) was administered to 86% of patients. Recurrence was recorded in 12 cases (5.2%). Surgical management was primarily open cystectomy (*n* = 187, 80.6%), while laparoscopic techniques were employed in 45 cases (19.4%), mainly in the later years of the study.

### 3.5. Associations Between Demographic and Clinical Variables

**Figure 4:**
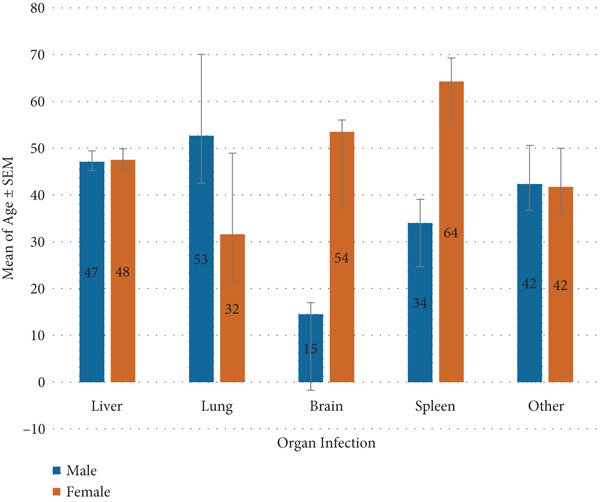
Comparison of mean age by organ involvement and gender.

The relationship between organ involvement and gender was not statistically significant (*χ* ^2^ = 0.55, *p* = 0.46; Figure 4).

**Figure 5:**
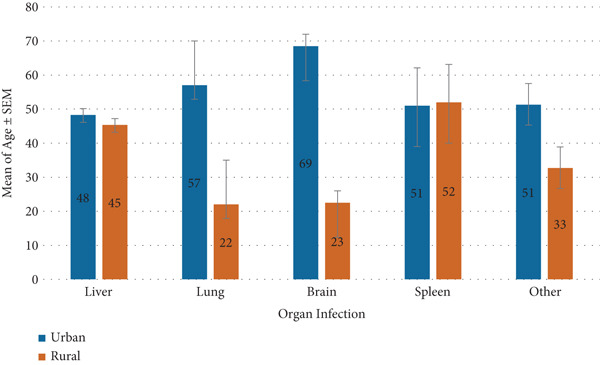
Comparison of mean age by organ involvement and residency (urban vs. rural).

A statistically significant association was found between residency and organ involvement in patients with CE (*χ* ^2^ = 5.78, df = 1, *p* = 0.016). Hepatic involvement was more frequent among urban residents (86.0%) compared with rural residents (73.2%), whereas extrahepatic cases were relatively more common in rural areas (26.8% vs. 14.0%) (Figure 5).

Similarly, a statistically significant association was observed between marital status and organ involvement in patients with CE (*χ* ^2^ = 6.77, df = 1, *p* = 0.009). Hepatic involvement was more frequent among married individuals (84.3%) compared with single patients (65.7%), whereas extrahepatic cases were relatively more common among single individuals (34.3% vs. 15.7%).

Marital status (OR = 2.54, 95% CI: 1.13–5.72, *p* = 0.024) and residency (OR = 0.48, 95% CI: 0.24–0.95, *p* = 0.035) were independently associated with hepatic echinococcosis.

Married individuals were more likely to have hepatic involvement, whereas those living in urban areas had a higher likelihood of hepatic echinococcosis compared with rural residents. No significant interaction was found between marital status and residency.

## 4. Discussion

This 17‐year retrospective cross‐sectional study provides the most comprehensive long‐term epidemiological assessment of surgically confirmed human CE in northeastern Iran to date. The findings offer valuable insights into the demographic and clinical characteristics, spatial heterogeneity, and temporal dynamics of CE in an endemic region characterized by close human–animal contact and intensive livestock farming.

Previous studies across Iran have reported considerable regional variation in the annual incidence of surgically treated CE, ranging from 0.74 to 3.27 per 100,000 population. In Fars Province, over a 15‐year period, the mean annual incidence was 0.74 per 100,000 [17]. In Hamedan, this rate was 1.5 per 100,1000 [18], and in West Azerbaijan it was approximately 0.98 per 100,000 [19]. In central Iran, the incidence has been reported at 1.16 per 100,000 [20]. In northeastern Iran, the highest previously reported incidence was observed in Sarakhs County (3.27 per 100,000) [21], whereas in the present study, Zaveh County recorded the highest rate (4.2 per 100,000). This elevated rate may reflect local differences in animal husbandry practices, dog ownership patterns, public health awareness, and access to healthcare services [22]. Notably, Zaveh—here approximately 80% of the population is rural and livestock farming is extensive—may facilitate sustained transmission of *E. granulosus* and, consequently, human CE. This elevated risk is likely driven by closer human–animal interactions and potential shortcomings in animal‐health and veterinary control measures. High CE prevalence has frequently been reported in rural populations in Iran; however, specific prevalence estimates should be cited here [23–25].

Comparatively, the annual incidence of surgically treated CE has been reported to be 13.1 per 100,000 in Kyrgyzstan and 10 per 100,000 in Peru, indicating substantially higher rates than those observed in Iran and in the present study [26, 27]. In contrast, reports from Europe show a markedly lower incidence of approximately 0.2 per 100,000 population [28].

Temporal trends revealed marked fluctuations in the incidence of surgically treated CE, with notable peaks in 2007, 2008, and 2015, followed by a general decline and stabilization after 2017. Similarly, in Uzbekistan, the average annual incidence of CE surgery was reduced by approximately 50% between 2011 and 2018 [29]. In Kazakhstan, the incidence decreased from 5.6 to 4.7 per 100,000 population between 2007 and 2016 [30]. These findings are also consistent with reports from various regions of Iran, where fluctuations and relative declines in the incidence of CE surgery have been observed in recent years [17, 31]. Such temporal variations may be attributed to improvements in diagnostic accuracy, modifications in referral and reporting systems, or differences in disease surveillance intensity over time. The downward trend in recent years may further reflect the impact of enhanced public health interventions, expanded community awareness, and changes in livestock management and slaughtering practices [5, 32].

Consistent with global patterns [33], the liver was the most commonly affected organ (81.5%), with abdominal pain being the predominant presenting symptom [34]. This finding aligns with several national studies in Iran, which also report liver involvement in the majority of cases [23, 25, 35]. Internationally, liver predominance is similarly reported, though the exact prevalence varies between regions, likely reflecting differences in transmission dynamics, diagnostic practices, and population characteristics [14, 34]. Kidney, brain, and spleen involvement were infrequent in our cohort, consistent with both national and global reports [36, 37]. Multi‐organ cases, although rare (4.7%), highlight the complexity and diagnostic challenges of CE in atypical presentations [37, 38]. The predominance of liver involvement is likely due to its role as the primary filter in portal circulation, a well‐documented pathophysiological feature of the disease [39]. Notably, compared with some studies reporting slightly higher splenic or pulmonary involvement [11, 37, 38], our findings suggest a relatively lower frequency of extrahepatic localization in this population, which could be influenced by referral patterns or regional epidemiology.

The female predominance observed in this study aligns with prior Iranian and global reports, possibly reflecting sociocultural and behavioral factors that increase women′s exposure to contaminated environments [40, 41]. The bimodal age distribution, with peaks among young adults (21–40 years) and the elderly (≥ 61 years), suggests both occupational exposure and long‐term cumulative infection risk. Kohansal et al. reported a high prevalence of infection among women aged 21–40 years in patients from Zanjan [42]. Similarly, a study conducted in southern Iran found a higher infection rate in individuals over 50 years of age [17]. This pattern may reflect cumulative exposure in older adults alongside occupational or behavioral risk factors in younger populations, particularly women involved in domestic livestock care in rural or suburban settings [43, 44].

The multivariate analysis revealed two independent associations: Married individuals and urban residents had a higher likelihood of hepatic involvement. The marital association may be explained by differences in lifestyle and exposure patterns, including greater participation in food preparation or household livestock handling [45]. Furthermore, urban residency was associated with increased hepatic involvement compared with rural residency. However, this could be attributed to better access to tertiary care centers and diagnostic facilities in urban areas, where surgical cases are therefore more likely to be detected and reported. Additionally, the practice of keeping dogs as household pets in urban settings could increase the risk of transmission to their owners [46].

Although the organ‐specific mean age differences were not statistically significant, they may indicate potential variations in disease progression or the time to clinical manifestation depending on the cyst location. For instance, pulmonary CE cases tended to occur in slightly younger individuals, possibly due to the earlier onset of symptoms such as cough or chest pain, which may prompt more rapid clinical evaluation. Furthermore, the structural characteristics of lung tissue such as its loose parenchyma, high vascularity, and the presence of negative intrathoracic pressure may facilitate faster cyst growth and enlargement within the lungs [47].

## 5. Conclusion

This 17‐year epidemiological assessment demonstrates that CE remains a persistent public health concern in northeastern Iran, despite modest declines in surgical incidence over time. Hepatic involvement continues to predominate, particularly among married and urban residents. Spatial clustering in Zaveh and Torbat Heydariyeh districts highlights enduring transmission cycles linked to livestock–dog interactions and human exposure. Importantly, this long‐term analysis provides insight into how sustained control measures and socio‐environmental factors influence CE incidence and surgical burden, addressing key questions regarding the effectiveness of regional interventions and transmission dynamics. Sustained One Health strategies—including veterinary deworming, regulated slaughtering, community education, and GIS‐assisted surveillance—are essential to mitigate transmission and evaluate the impact of control efforts. Future population‐based studies integrating molecular, environmental, and behavioral data will be crucial for refining prevention strategies and reducing disease burden in endemic areas.

## Ethics Statement

The ethical considerations of this study were approved by the Ethics Committee of the Torbat Heydariyeh University of Medical Sciences (Ethical Code: IR.THUMS.REC.1398.028).

## Conflicts of Interest

The authors declare no conflicts of interest.

## Funding

The Vice Chancellor of Research at Torbat Heydariyeh University of Medical Sciences supported this research (Grant Number: SRC‐98‐137).

## Data Availability

The data that support the findings of this study are available from the corresponding author upon reasonable request.

## References

[1] Mohaghegh M. A. , Yousefi-Darani H. , Azami M. , Ghomashlooyan M. , Hashemi N. , Jabalameli Z. , Mahdavi M. , McManus D. P. , and Hejazi S. H. , Analysis of the COX1 Gene in *Echinococcus granulosus* From Sheep in Northeast Iran Using PCR High-Resolution Melting (qPCR-HRM) Curve Analysis, Tropical Biomedicine. (2018) 35, no. 1, 91–99, 33601781. 33601781

[2] Mohaghegh M. A. , Yousofi-Darani H. , Jafarian A. H. , Mirbadie S. R. , Fasihi-Harandi M. , Ghavimi R. , Jabalameli Z. , Azami M. , Mohammadi M. , and Hejazi S. H. , Isolated Human and Livestock *Echinococcus granulosus* Genotypes Using Real-Time PCR of COX1 Gene in Northeast Iran, Acta Parasitologica. (2019) 64, no. 3, 679–685, 10.2478/s11686-019-00117-w, 2-s2.0-85074022040. 31538303

[3] Rawat S. , Kumar R. , Raja J. , Singh R. S. , and Thingnam S. K. S. , Pulmonary Hydatid Cyst: Review of Literature, Journal of Family Medicine and Primary Care. (2019) 8, no. 9, 2774–2778, 10.4103/jfmpc.jfmpc_624_19. PMC6820383 31681642

[4] Mirbadie S. , Zivdari M. , Kalani H. , Vafaei M. , Izadi S. , Jabalameli Z. , Mohammadi M. , Yadagiri G. , Heydarian P. , Mirzaei F. , and Mohaghegh M. A. , Molecular Identification of *Echinococcus granulosus* Sensu Lato by Mitochondrial COX1 and SSU-rDNA Markers in Dogs in the West of Iran, Gene Reports. (2020) 19, 100616, 10.1016/j.genrep.2020.100616.

[5] Wen H. , Vuitton L. , Tuxun T. , Li J. , Vuitton D. A. , Zhang W. , and McManus D. P. , Echinococcosis: Advances in the 21st Century, Clinical Microbiology Reviews. (2019) 32, no. 2, 10.1128/CMR.00075-18, 2-s2.0-85061583690, 30760475. PMC6431127 30760475

[6] Sokouti M. , Sadeghi R. , Pashazadeh S. , Abadi S. E. H. , Sokouti M. , Rezaei-Hachesu P. , Ghojazadeh M. , and Sokouti B. , A Systematic Review and Meta-Analysis on the Treatment of Liver Hydatid Cyst: Comparing Laparoscopic and Open Surgeries, Arab Journal of Gastroenterology. (2017) 18, no. 3, 127–135, 10.1016/j.ajg.2017.09.010, 2-s2.0-85030642724. 28988788

[7] Botezatu C. , Mastalier B. , and Patrascu T. , Hepatic Hydatid Cyst—Diagnose and Treatment Algorithm, Journal of Medicine and Life. (2018) 11, no. 3, 203–209, 10.25122/jml-2018-0045, 2-s2.0-85057113702, 30364592. 30364592 PMC6197524

[8] Yuan W. H. , Lee R. C. , Chou Y. H. , Chiang J. H. , Chen Y. K. , and Hsu H. C. , Hydatid Cyst of the Liver: A Case Report and Literature Review, Kaohsiung Journal of Medical Sciences. (2005) 21, no. 9, 418–423, 10.1016/S1607-551X(09)70144-5. 16248126 PMC11917981

[9] McManus D. P. , Gray D. J. , Zhang W. , and Yang Y. , Diagnosis, Treatment, and Management of Echinococcosis, BMJ. (2012) 344, e3866, 10.1136/bmj.e3866, 2-s2.0-84862505336. 22689886

[10] Aziz H. , Seda P. , Aswani Y. , Gosse M. D. , Krishnakumari A. J. , and Pawlik T. M. , Cystic Echinococcosis of the Liver, Journal of Gastrointestinal Surgery. (2025) 29, no. 3, 101974, 10.1016/j.gassur.2025.101974. 39864780

[11] Shahriarirad R. , Erfani A. , Ebrahimi K. , Rastegarian M. , Eskandarisani M. , Ziaian B. , and Sarkari B. , Hospital-Based Retrospective Analysis of 224 Surgical Cases of Lung Hydatid Cyst From Southern Iran, Journal of Cardiothoracic Surgery. (2023) 18, no. 1, 10.1186/s13019-023-02327-w, 37400848. PMC10316629 37400848

[12] Şahin S. and Kaya B. , Evaluation of Hydatid Cyst Cases: A Single-Center Retrospective Study, Türkiye Parazitolojii Dergisi. (2025) 48, no. 4, 222–227, 10.4274/tpd.galenos.2024.79553, 39844608. 39844608

[13] Govindasamy A. , Bhattarai P. R. , and John J. , Liver Cystic Echinococcosis: A Parasitic Review, Therapeutic Advances in Infectious Disease. (2023) 10, 20499361231171478, 10.1177/20499361231171478. 37197609 PMC10184195

[14] Nunnari G. , Pinzone M. R. , Gruttadauria S. , Celesia B. M. , Madeddu G. , Malaguarnera G. , Pavone P. , Cappellani A. , and Cacopardo B. , Hepatic Echinococcosis: Clinical and Therapeutic Aspects, World Journal of Gastroenterology. (2012) 18, no. 13, 1448–1458, 10.3748/wjg.v18.i13.1448, 2-s2.0-84859334829, 22509076. 22509076 PMC3319940

[15] Khalkhali H. R. , Foroutan M. , Khademvatan S. , Majidiani H. , Aryamand S. , Khezri P. , and Aminpour A. , Prevalence of Cystic Echinococcosis in Iran: A Systematic Review and Meta-Analysis, Journal of Helminthology. (2018) 92, no. 3, 260–268, 10.1017/S0022149X17000463, 2-s2.0-85020246517, 28589871. 28589871

[16] Fasihi Harandi M. , Budke C. M. , and Rostami S. , The Monetary Burden of Cystic Echinococcosis in Iran, PLoS Neglected Tropical Diseases. (2012) 6, no. 11, e1915, 10.1371/journal.pntd.0001915, 2-s2.0-84870766455, 23209857. 23209857 PMC3510083

[17] Shahriarirad R. , Erfani A. , Eskandarisani M. , Rastegarian M. , Taghizadeh H. , and Sarkari B. , Human Cystic Echinococcosis in Southwest Iran: A 15-Year Retrospective Epidemiological Study of Hospitalized Cases, Tropical Medicine and Health. (2020) 48, no. 1, 10.1186/s41182-020-00238-3, 32577086. PMC7304208 32577086

[18] Fallah N. , Rahmati K. , and Fallah M. , Prevalence of Human Hydatidosis Based on Hospital Records in Hamadan West of Iran From 2006 to 2013, Iranian Journal of Parasitology. (2017) 12, no. 3, 453–460, 28979357. 28979357 PMC5623927

[19] Hajipirloo H. M. , Bozorgomid A. , Alinia T. , Tappeh K. H. , and Mahmodlou R. , Human Cystic Echinococcosis in West Azerbaijan, Northwest Iran: A Retrospective Hospital Based Survey From 2000 to 2009, Iranian Journal of Parasitology. (2013) 8, no. 2, 323–326, 23914247. 23914247 PMC3724159

[20] Farazi A. , Zarinfar N. , Kayhani F. , and Khazaie F. , Hydatid Disease in the Central Region of Iran: A 5-Year Epidemiological and Clinical Overview, Central Asian Journal of Global Health. (2019) 8, no. 1, 10.5195/cajgh.2019.364, 32002314. PMC6948357 32002314

[21] Ebrahimipour M. , Budke C. M. , Najjari M. , Cassini R. , and Asmarian N. , Bayesian Spatial Analysis of the Surgical Incidence Rate of Human Cystic Echinococcosis in North-Eastern Iran, Acta Tropica. (2016) 163, 80–86, 10.1016/j.actatropica.2016.08.003, 2-s2.0-84982813803, 27496620. 27496620

[22] Hejazi S. H. , Mirbadie S. R. , Jafari R. , Rezaiemanesh M. R. , Azizi O. , Badmasti F. , Kalani H. , Cheraghipour K. , Heydarian P. , Hashemi N. , Izadi S. , Jabalameli Z. , and Mohaghegh M. A. , *Echinococcus Granulosus* Sheep Strain (G1) as the Predominant Genotype in Definitive Host (Dogs) Isolates in Northeastern Iran, Veterinary Parasitology: Regional Studies and Reports. (2024) 48, 100975, 10.1016/j.vprsr.2023.100975. 38316501

[23] Haddad M. H. F. , Sepahvand Z. , Fadaei T. , and Belali R. , Epidemiological Characteristics of Human Cystic Echinococcosis in Khuzestan Province (Iran), 2011-2021: A Retrospective Analytical Study, Journal of Parasitic Diseases. (2023) 47, no. 4, 718–726, 10.1007/s12639-023-01619-1. 38009155 PMC10667199

[24] Chalechale A. , Hashemnia M. , Rezaei F. , and Sayadpour M. , *Echinococcus granulosus* in Humans Associated With Disease Incidence in Domestic Animals in Kermanshah, West of Iran, Journal of Parasitic Diseases. (2016) 40, no. 4, 1322–1329, 10.1007/s12639-015-0681-1, 2-s2.0-84926030507, 27876940. 27876940 PMC5118307

[25] Ghabouli Mehrabani N. , Kousha A. , Khalili M. , Mahami Oskouei M. , Mohammadzadeh M. , Alizadeh S. , Maleksabet A. , and Hamidi F. , Hydatid Cyst Surgeries in Patients Referred to Hospitals in East Azerbaijan Province During 2009–2011, Iranian Journal of Parasitology. (2014) 9, no. 2, 233–238, 25848390. 25848390 PMC4386044

[26] Paternoster G. , Boo G. , Wang C. , Minbaeva G. , Usubalieva J. , Raimkulov K. M. , Zhoroev A. , Abdykerimov K. K. , Kronenberg P. A. , Müllhaupt B. , Furrer R. , Deplazes P. , and Torgerson P. R. , Epidemic Cystic and Alveolar Echinococcosis in Kyrgyzstan: An Analysis of National Surveillance Data, Lancet Global Health. (2020) 8, no. 4, e603–e611, 10.1016/S2214-109X(20)30038-3, 32199126. 32199126

[27] Deplazes P. , Rinaldi L. , Alvarez Rojas C. A. , Torgerson P. R. , Harandi M. F. , Romig T. , Antolova D. , Schurer J. M. , Lahmar S. , Cringoli G. , Magambo J. , Thompson R. C. , and Jenkins E. J. , Global Distribution of Alveolar and Cystic Echinococcosis, Advances in Parasitology. (2017) 95, 315–493, 10.1016/bs.apar.2016.11.001, 2-s2.0-85009801529. 28131365

[28] The European Union Summary Report on Trends and Sources of Zoonoses, Zoonotic Agents and Food-Borne Outbreaks in 2016, EFSA Journal. (2017) 15, no. 12, e05077, 10.2903/j.efsa.2017.5077. 32625371 PMC7009962

[29] Colpani A. , Achilova O. , D′Alessandro G. L. , Budke C. M. , Mariconti M. , Muratov T. , Vola A. , Mamedov A. , Giordani M. T. , Urukov X. , De Silvestri A. , Suvonkulov U. , Brunetti E. , and Manciulli T. , Trends in the Surgical Incidence of Cystic Echinococcosis in Uzbekistan From 2011 to 2018, American Journal of Tropical Medicine and Hygiene. (2021) 106, no. 2, 724–728, 10.4269/ajtmh.21-0261, 34902836. 34902836 PMC8832901

[30] Mustapayeva A. , Manciulli T. , Zholdybay Z. , Juskiewicz K. , Zhakenova Z. , Shapiyeva Z. , Medetov Z. , Vola A. , Mariconti M. , Brunetti E. , Budke C. M. , Khalykova M. , and Duisenova A. , Incidence Rates of Surgically Managed Cystic Echinococcosis in Kazakhstan, 2007–2016, American Journal of Tropical Medicine and Hygiene. (2020) 102, no. 1, 90–95, 10.4269/ajtmh.19-0572, 31802731. 31802731 PMC6947765

[31] Yazdani A. , Familsatarian B. , Bagheri H. , Mohammadzadeh A. , and Hosseinkhani Z. , A 10-Zear Epidemiological Study of Human Cystic Echinococcosis in Qazvin Province, Iran, Journal of Inflammatory Diseases. (2021) 25, no. 3, 183–190, 10.32598/JQUMS.25.3.3.

[32] Hajjafari A. , Sadr S. , Santucciu C. , Masala G. , Bayat M. , Lotfalizadeh N. , Borji H. , Partovi Moghaddam S. , and Hajjafari K. , Advances in Detecting Cystic Echinococcosis in Intermediate Hosts and New Diagnostic Tools: A Literature Review, Veterinary Sciences. (2024) 11, no. 6, 10.3390/vetsci11060227. PMC11209443 38921974

[33] Mihmanli M. , Idiz U. O. , Kaya C. , Demir U. , Bostanci O. , Omeroglu S. , and Bozkurt E. , Current Status of Diagnosis and Treatment of Hepatic Echinococcosis, World Journal of Hepatology. (2016) 8, no. 28, 1169–1181, 10.4254/wjh.v8.i28.1169, 2-s2.0-84994159053, 27729953. 27729953 PMC5055586

[34] Govindasamy A. , Bhattarai P. R. , Van Niekerk J. , and John J. , Liver Cystic Echinococcosis: A Retrospective Study on the Demographics and Clinical Profile of Patients Managed at a Single Tertiary Institution in Central Eastern Cape Province, South Africa, South African Medical Journal. (2024) 114, no. 5, e2195, 10.7196/SAMJ.2024.v114i5.2195, 39041470. 39041470

[35] Erfani A. , Shahriarirad R. , Eskandarisani M. , Rastegarian M. , and Sarkari B. , Management of Liver Hydatid Cysts: A Retrospective Analysis of 293 Surgical Cases From Southern Iran, Journal of Tropical Medicine. (2023) 2023, 9998739, 10.1155/2023/9998739. 37377601 PMC10292944

[36] Shahriarirad R. , Erfani A. , Eskandarisani M. , Rastegarian M. , and Sarkari B. , Uncommon Locations of Cystic Echinococcosis: A Report of 46 Cases From Southern Iran, Surgery Research and Practice. (2020) 2020, 2061045, 10.1155/2020/2061045. 33015320 PMC7520003

[37] Harizanov R. N. , Rainova I. G. , and Kaftandjiev I. T. , Extra-Hepatopulmonary Cystic Echinococcosis in Bulgaria: Frequency, Management and Outcome of the Disease, Parasitology. (2021) 148, no. 5, 562–565, 10.1017/S0031182020002206. 33213598 PMC10950371

[38] Mahmoudi S. , Dolatzadeh M. , Manzari Tavakoli G. , Pourakbari B. , Abdolsalehi M. R. , and Mamishi S. , Pediatric Cystic Echinococcosis in Tehran, Iran: A 9-Year Retrospective Epidemiological and Clinical Survey of Hospitalized Cases, Foodborne Pathogens and Disease. (2024) 21, no. 10, 662–668, 10.1089/fpd.2024.0034. 39119694

[39] Mihai C. M. , Lupu A. , Chisnoiu T. , Balasa A. L. , Baciu G. , Lupu V. V. , Popovici V. , Suciu F. , Enache F. D. , Cambrea S. C. , and Stoicescu R. M. , A Comprehensive Analysis of *Echinococcus granulosus* Infections in Children and Adolescents: Results of a 7-Year Retrospective Study and Literature Review, Pathogens. (2025) 14, no. 1, 10.3390/pathogens14010053, 39861014. PMC11768134 39861014

[40] Khatonaki H. , Mazaherifar S. , Shokoohi G. , Hatami N. , Vafai Z. , Javdani F. , and Abolghazi A. , The Epidemiology and Medical Care Costs of *Echinococcus granulosusis* in Jahrom, Southern Iran From 2007 to 2017, Infection Ecology & Epidemiology. (2020) 10, no. 1, 1821503, 10.1080/20008686.2020.1821503, 33062216. 33062216 PMC7534386

[41] Ma T. , Wang Q. , Hao M. , Xue C. , Wang X. , Han S. , Wang Q. , Zhao J. , Ma X. , Wu X. , Jiang X. , Cao L. , Yang Y. , Feng Y. , Gongsang Q. , Scheffran J. , Fang L. , Maude R. J. , Zheng C. , Ding F. , Wu W. , and Jiang D. , Epidemiological Characteristics and Risk Factors for Cystic and Alveolar Echinococcosis in China: An Analysis of a National Population-Based Field Survey, Parasites & Vectors. (2023) 16, no. 1, 10.1186/s13071-023-05788-z. PMC10239570 37270512

[42] Kohansal M. H. , Nourian A. , and Bafandeh S. , Human Cystic Echinococcosis in Zanjan Area, Northwest Iran: A Retrospective Hospital Based Survey Between 2007 and 2013, Iranian Journal of Public Health. (2015) 44, no. 9, 1277–1282, 26587503. 26587503 PMC4645786

[43] Acosta-Jamett G. , Hernández F. A. , Castro N. , Tamarozzi F. , Uchiumi L. , Salvitti J. C. , Cueva M. , and Casulli A. , Prevalence Rate and Risk Factors of Human Cystic Echinococcosis: A Cross-Sectional, Community-Based, Abdominal Ultrasound Study in Rural and Urban North-Central Chile, PLoS Neglected Tropical Diseases. (2022) 16, no. 3, e0010280, 10.1371/journal.pntd.0010280, 35263331. 35263331 PMC8936472

[44] Kakamad F. H. , Anwar K. A. , Ahmed H. K. , Habibullah I. J. , Kaka Ali H. H. , Nasralla H. A. , Abdullah H. O. , Tahir S. H. , Kareem H. O. , Hasan A. H. , Gharib D. T. , Asaad H. R. , Mohammed A. A. , Abdalla B. A. , Esmaeil D. A. , Rashid R. J. , and Hamahussein K. F. , Risk Factors Associated With Human Echinococcosis: A Systematic Review and Meta-Analysis, Frontiers in Veterinary Science. (2024) 11, 1480579, 10.3389/fvets.2024.1480579, 39654835. 39654835 PMC11625768

[45] Uchiumi L. , Mujica G. , Araya D. , Salvitti J. C. , Sobrino M. , Moguillansky S. , Solari A. , Blanco P. , Barrera F. , Lamunier J. , Arezo M. , Seleiman M. , Yadon Z. E. , Tamarozzi F. , Casulli A. , and Larrieu E. , Prevalence of Human Cystic Echinococcosis in the Towns of Ñorquinco and Ramos Mexia in Rio Negro Province, Argentina, and Direct Risk Factors for Infection, Parasites & Vectors. (2021) 14, no. 1, 10.1186/s13071-021-04753-y, 34011406. PMC8136178 34011406

[46] Hejazi S. H. , Kalantari R. , Mousavi S. M. , Safari M. , Ghayour Z. , Nokhodian Z. , and Esmaeilifallah M. , Seroprevalence of Human Cystic Echinococcosis in Individuals Occupationally Exposed to Canidae in Central Iran: A Case-Control Study, Food and Waterborne Parasitology. (2025) 39, e00263, 10.1016/j.fawpar.2025.e00263, 40330839. 40330839 PMC12051609

[47] Sarkar M. , Pathania R. , Jhobta A. , Thakur B. R. , and Chopra R. , Cystic Pulmonary Hydatidosis, Lung India. (2016) 33, no. 2, 179–191, 10.4103/0970-2113.177449, 2-s2.0-84960347812. 27051107 PMC4797438

